# Risk of first cervical HPV infection and pre-cancerous lesions after onset of sexual activity: analysis of women in the control arm of the randomized, controlled PATRICIA trial

**DOI:** 10.1186/s12879-014-0551-y

**Published:** 2014-10-30

**Authors:** Xavier Castellsagué, Jorma Paavonen, Unnop Jaisamrarn, Cosette M Wheeler, S Rachel Skinner, Matti Lehtinen, Paulo Naud, Song-Nan Chow, Maria Rowena Del Rosario-Raymundo, Julio C Teixeira, Johanna Palmroth, Newton S de Carvalho, Maria Julieta V Germar, Klaus Peters, Suzanne M Garland, Anne Szarewski, Willy AJ Poppe, Barbara Romanowski, Tino F Schwarz, Wiebren AA Tjalma, F Xavier Bosch, Marie-Cecile Bozonnat, Frank Struyf, Gary Dubin, Dominique Rosillon, Laurence Baril, for the HPV PATRICIA Study Group

**Affiliations:** Unit of Infections and Cancer, Cancer Epidemiology Research Program, Institut Català d'Oncologia (ICO), IDIBELL, CIBER-ESP, L'Hospitalet de Llobregat, Avda. Gran via 199-203, 08908 L'Hospitalet de Llobregat, Barcelona, Catalonia Spain; Department of Obstetrics and Gynaecology, University of Helsinki, Helsinki, Finland; Department of Obstetrics and Gynaecology, Faculty of Medicine, Chulalongkorn University, Bangkok, Thailand; Departments of Pathology and Obstetrics and Gynecology, University of New Mexico Health Sciences Center, Albuquerque, NM USA; Vaccines Trials Group, Telethon Institute for Child Health Research, Perth, WA and Sydney University Discipline of Paediatrics and Child Health, Children's Hospital Westmead, Sydney, NSW Australia; University of Tampere, School of Public Health, Tampere, Finland; Department of Gynecology & Obstetrics Federal University of Rio Grande do Sul - UFRGS/HCPA, Hospital de Clínicas de Porto Alegre, Porto Alegre, Brazil; Department of Obstetrics and Gynecology, College of Medicine and the Hospital, National Taiwan University, Taipei, Taiwan; San Pablo Colleges Medical Center, San Pablo City, Laguna, Philippines; Departamento de Tocoginecologia da Unicamp, University of Campinas, Campinas, Sao Paulo Brazil; Department of Obstetrics and Gynecology, Central Hospital of North Carelian, Joensuu, Finland; Department of Gynecology and Obstetrics, Infectious Diseases in Gynecology and Obstetrics Sector, Federal University of Paraná, Curitiba, Parana Brazil; Department of Obstetrics and Gynaecology, University of the Philippines College of Medicine, Philippines General Hospital, Manila, Philippines; Facharzt für Frauenheilkunde und Geburtshilfe, Hamburg, Germany; Department of Microbiology and Infectious Diseases, The Royal Women's Hospital, Parkville/Department of Microbiology, The Royal Children's Hospital, Parkville/Murdoch Children's Research Institute, Parkville/Department of Obstetrics and Gynaecology, University of Melbourne, Parkville, Victoria Australia; Centre for Cancer Prevention, Wolfson Institute of Preventive Medicine, Queen Mary University of London, London, UK; Department of Gynaecology, University Hospital KU Leuven Gasthuisberg, Leuven, Belgium; Division of Infectious Diseases, Department of Medicine, Faculty of Medicine and Dentistry, University of Alberta, Edmonton, AB Canada; Central Laboratory and Vaccination Centre, Stiftung Juliusspital, Academic Teaching Hospital of the University of Wuerzburg, Wuerzburg, Germany; Multidisciplinary Breast Clinic and Gynaecological Oncology, Antwerp University Hospital, University of Antwerp, Antwerpen, Belgium; Network on Cooperative Cancer Research, RTICC, Catalonia, Spain; 4Clinics, Paris, France; GlaxoSmithKline Vaccines, Wavre, Belgium; GlaxoSmithKline Vaccines, King of Prussia, PA USA

**Keywords:** HPV, CIN, Sexual intercourse, Time, Risk

## Abstract

**Background:**

More information is needed about time between sexual initiation and human papillomavirus (HPV) infection and development of cervical precancer.

**Methods:**

The objectives were to investigate the time between first sexual activity and detection of first cervical HPV infection or development of first cervical intraepithelial neoplasia (CIN), and associated factors in women from the double-blind, multinational, 4-year PATRICIA trial. PATRICIA enroled women aged 15-25 years with no more than 6 lifetime sexual partners. Women were randomized 1:1 to the HPV-16/18 AS04-adjuvanted vaccine or to control, but only women from the control arm who began sexual intercourse during the study or within 6 months before enrolment, and had no HPV infection detected before the recorded date of their first sexual intercourse, were included in the present analysis. The time between onset of sexual activity and detection of the first cervical HPV infection or development of the first CIN lesion was analyzed using Kaplan-Meier and univariate and multivariable Cox proportional-hazards models.

**Results:**

A total of 9337 women were enroled in the control arm of PATRICIA of whom 982 fulfilled the required inclusion criteria for analysis. A cumulative total of 28%, 44%, and 62% of the subjects had HPV infection within 12, 24, and 48 months, respectively. The overall incidence rate was 27.08 per 100 person-years. The most common oncogenic types associated with 6-month persistent infection were HPV-16 (incidence rate: 2.74 per 100 person-years), HPV-51 (2.70), HPV-52 (1.66), HPV-66 (1.14), and HPV-18 (1.09). Increased infection risk was associated with more lifetime sexual partners, being single, *Chlamydia trachomatis* history, and duration of hormone use. CIN1+ and CIN2+ lesions were most commonly associated with HPV-16, with an overall incidence rate of 1.87 and 1.07 per 100 person-years, respectively. Previous cervical HPV infection was most strongly associated with CIN development.

**Conclusions:**

More than 25% of women were infected with HPV within 1 year of beginning sexual activity. Without underestimating the value of vaccination at older ages, our findings emphasize its importance before sexual initiation.

**Trial registration:**

clinicaltrials.gov: NCT00122681.

**Electronic supplementary material:**

The online version of this article (doi:10.1186/s12879-014-0551-y) contains supplementary material, which is available to authorized users.

## Background

Human papillomavirus (HPV), the causal agent of cervical cancer, is believed to be the most common sexually transmitted infection worldwide, with up to 75% of sexually active people being infected during their life [1]. A persistent infection with an oncogenic HPV type is almost always required before development of cervical pre-cancer or cancer [2]-[4]. Fortunately, most HPV infections are transient and clear naturally within a few months [5]. Individual immune responses are important in determining the path of an infection, and a limited number of other non-viral determinants with modest risks for HPV infection and progression have been well established [6]-[11].

HPV vaccination programs have been introduced in many countries worldwide and are primarily targeted at adolescent girls. Vaccination before first sexual intercourse is important because HPV infection is frequently detected in sexually active adolescent girls and young women [12],[13]. HPV infection often occurs shortly after onset of sexual activity, and many girls and women acquire an infection with their first sexual relationship [13]-[16].

To add to the body of knowledge concerning the natural history of HPV, more data are needed about the time between onset of sexual activity and first acquisition of HPV infection and first development of detectable lesions under surveillance using defined referral algorithms. However, few prospective data are available [13],[15],[16]. The control arms of large trials of prophylactic HPV vaccines are well placed to inform such questions, with large samples of women with relatively diverse behavioral characteristics, and data collected on acquisition of different HPV types, development of pre-cancerous lesions, and factors potentially influencing infection and progression to the first pre-cancerous lesion.

We have previously reported an analysis of the risk of progression from cervical HPV infection to pre-cancerous lesions or clearance of infection from the PApilloma TRial against Cancer In young Adults (PATRICIA), a phase III trial of the HPV-16/18 AS04-adjuvanted vaccine (*Cervarix®*) in over 18,000 young women [17]. Here, we report an analysis of the time between first sexual intercourse and acquisition of the first cervical HPV infection or development of the first pre-cancerous lesion.

## Methods

This analysis was based on data obtained from the control arm of the double-blind, randomized, multinational (14 countries), controlled, 4-year PATRICIA trial which enrolled women aged 15-25 years. The objectives were to investigate the time between first sexual activity and either detection of the first cervical HPV infection or development of the first cervical intraepithelial neoplasia (CIN), and associated factors.

### Study population and procedures

The PATRICIA trial design has been described previously [18],[19]. Written informed consent to participate in the clinical trial was obtained from all adult participants; for minors, written informed consent was obtained from their parents or guardians and assent from the participants themselves.The protocol and other materials were approved by local independent ethics committees or institutional review boards (Additional file 1). The clinical trial was carried out in accordance with The Code of Ethics of the World Medical Association (Declaration of Helsinki) and is registered at ClinicalTrials.gov under number NCT00122681.

PATRICIA enrolled women with no more than 6 lifetime sexual partners. The exclusion criterion of no more than six lifetime sexual partners was not applied in Finland, in accordance with local regulatory and ethical requirements, so women with more than six lifetime sexual partners enrolled in Finland were included in the trial [20]. The analysis was based on women from the control arm of the total vaccinated cohort for efficacy (TVC-E) which included all women who received at least one dose of control vaccine and had normal or low grade cytology at baseline. The present analysis included women who began sexual intercourse either during the follow-up period or less than 6 months before enrolment. Women beginning sexual intercourse during the study must not have had HPV infection detected before the first recorded date of sexual intercourse. However, women who began sexual intercourse within 6 months before enrolment were included regardless of whether they had a prevalent HPV infection at baseline or not.HPV genotyping of cervical liquid-based cytology samples by polymerase chain reaction (PCR) was done at baseline and every 6 months. The Bethesda system was used for cytologic classification approximately every 12 months, with histologic classification of any biopsies. We tested for the following oncogenic HPV types: HPV-16, 18, 31, 33, 35, 39, 45, 51, 52, 56, 58, 59, 66, and 68, and for the following non-oncogenic types: HPV-6, 11, 34, 40, 42, 43, 44, 53, 54, 70, and 74 [21].

A self-administered behavioral questionnaire collecting data on smoking, sexual activity, and contraception was completed 1 month after first vaccination and yearly thereafter during the follow-up period [22]. Participants were informed that the term sexual intercourse included penetrative, genital-to-genital, or oral-genital sexual contact.

### Endpoint definitions

The first HPV infection detected or first CIN detected was used in the analysis. HPV infections were classified as an infection of any duration (HPVI) and as a 6-month persistent infection (6MPI). A 6MPI was defined as detection of the same HPV genotype in cervical samples at two consecutive evaluations over approximately a 6-month period i.e. a sequence of positive samples with the same HPV type not interrupted by negative samples over a total range of >5 months (>150 days). The start of the 6MPI was defined as the date of the first positive sample in the sequence. Histopathologically confirmed CIN was detected in biopsy samples after colposcopy or excision samples after treatment. Endpoints were defined as CIN grade 1 or greater (CIN1+) and CIN grade 2 or greater (CIN2+). Only HPV-positive lesions were included in the analysis.

### Statistical analysis

Incidence rates for HPVI, 6MPI and CIN were calculated per 100 person-years (PY) according to individual HPV type, with 95% confidence intervals (CI). The time to detection of the first cervical HPV infection or development of the first CIN lesion was measured from the date of first sexual intercourse reported by the woman. Thus, although visits were scheduled for every 6 months, it was possible to establish a duration of infection of less than 6 months. The time between onset of sexual activity and detection of the first infection or CIN lesion was analysed using the Kaplan-Meier method as well as univariate and multivariable Cox proportional-hazards models. The statistical unit was the subject.

Nine potential factors (covariates) associated with acquisition of HPV infection were included in the models: region, tobacco exposure measured as number of pack-years (one pack-year was equivalent to 365 packs of cigarettes), age at first sexual intercourse (15-17 years and ≥18 years), number of sexual partners during the past 12 months, number of lifetime sexual partners, history of *Chlamydia trachomatis* during the past 12 months, marital/partner status, use of hormones for contraception or other indication, and duration of use of hormones for contraception or other indication. The number of lifetime sexual partners was determined from the behavioral questionnaire. All behavioural covariates were considered as time dependent and therefore no proportional hazard checks were required. Region and age were considered as time-fixed covariates and the hazard assumptions were assessed directly from the Kaplan-Meier curves. In addition, for the time to detection of a 6MPI, CIN1+ and CIN2+, the models also included previous cervical HPV infection as a time-varying covariate. Previous cervical HPV infection was defined as an HPV infection preceding the onset of the referent 6MPI or preceding the onset of the referent infection associated with a CIN1+ or CIN2 +.

Data were censored for women who completed the study at 54 months after first sexual intercourse (although follow-up lasted for 48 months, women were included in the analysis if they had their first sexual experience within 6 months before enrolment). For women who did not complete the study, data were censored at their last recorded visit. Covariates with a p-value <0.2 in the univariate model were included in the multivariable models, with the exception of region which was always included regardless of the p-value obtained. All analyses were performed using SAS version 9.2.

## Results

### Subject disposition and characteristics

**Figure 1:**
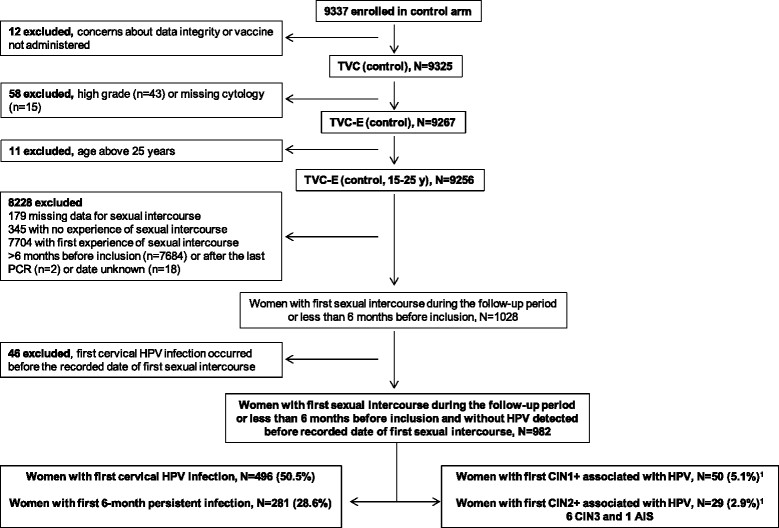
**Subject disposition.**

A total of 982 women who experienced first sexual intercourse during the study and had no HPV infection detected before the recorded date of their first experience of sexual intercourse, and women who experienced first sexual intercourse less than 6 months before inclusion, were included in the analysis (Figure 1). Among these 982 women, mean age was 17.4 years (standard deviation 2.13), and most were from Europe (65.6%, with 60.8% from Finland), followed by Asia-Pacific (17.1%), North America (9.6%) and Latin America (7.7%). Most women included in the present analysis had not experienced sexual intercourse before study entry (n = 793, 80.8%), 169 women (17.2%) had experienced first sexual intercourse within 6 months prior to study entry, and data on sexual history were missing for 20 women (2.0%). Eight (4.7%) and nine (5.3%) of the women who experienced first sexual intercourse prior to study entry were seropositive for HPV-16 or HPV-18, respectively, at study entry.

### First cervical HPV infection of any duration and 6-month persistent infection: incidence and association of covariates

**Figure 2:**
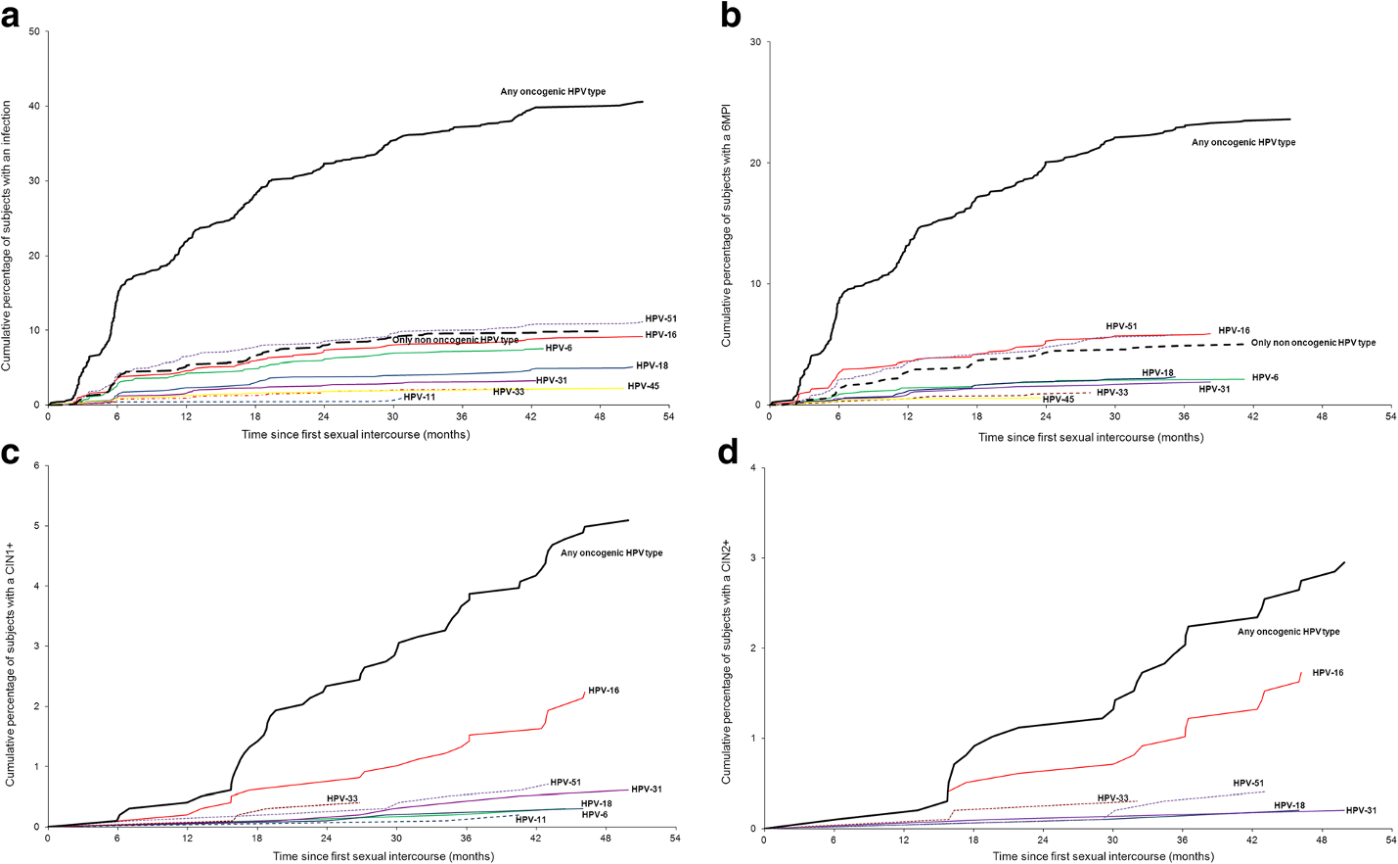
**Risk of developing a first cervical HPV infection or lesion following first sexual intercourse. a**. HPVI. **b**. 6MPI. **c**. CIN1+. **d**. CIN2+. Any oncogenic HPV type: HPV-16, 18, 31, 33, 35, 39, 45, 51, 52, 56, 58, 59, 66, and 68.

An HPVI was detected in 496 women (50.5%; 27.08 per 100 person-years) (Table 1). No HPVI had been detected by the end of the follow-up period in 446 women (45.4%) who completed the study, and no HPVI had been detected by the time of their last visit in 40 women (4.1%) who discontinued before the end of the study. Corresponding values for 6MPI were 281 (28.6%; 13.29 per 100 person-years), 648 (66.0%) and 53 (5.4%). The first cases of HPV infections were detected within 2-3 months after sexual initiation and the cumulative number of women with an infection increased progressively throughout follow-up for both HPVI and 6MPI (Figure 2a and b). The cumulative percentage of women infected with any HPV type at 12, 18, 24, 36 and 48 months was 28%, 36%, 44%, 53% and 62%, respectively (Figure 2a).

**Table 1:** **Incidence of first cervical HPVI, 6MPI, CIN1+ and CIN2+**

HPV type	HPVI (N = 496)		6MPI (N = 281)		CIN1+ (N = 50)		CIN2+ (N = 29)	
	n (%)	Incidence per 100 PY (95% CI) ^1^	n (%)	Incidence per 100 PY (95% CI) ^1^	n (%)	Incidence per 100 PY (95% CI) ^1^	n (%)	Incidence per 100 PY (95% CI) ^1^
All	496 (50.5)^2^	27.08 (24.75-29.57)	281 (28.6)^2^	13.29 (11.78-14.94)	50 (5.1)^2^	1.87 (1.39-2.46)	29 (3.0)^2^	1.07 (0.72-1.54)
**Oncogenic HPV types**								
16	90 (18.2)	4.91 (3.95-6.04)	58 (20.6)	2.74 (2.08-3.55)	22 (44.0)	0.82 (0.52-1.24)	17 (58.6)	0.63 (0.37-1.01)
18	50 (10.1)	2.73 (2.03-3.60)	23 (8.2)	1.09 (0.69-1.63)	3 (6.0)	0.11 (0.02-0.33)	2 (6.9)	0.07 (0.01-0.27)
31	32 (6.5)	1.75 (1.20-2.47)	19 (6.8)	0.90 (0.54-1.40)	6 (12.0)	0.22 (0.08-0.49)	2 (6.9)	0.07 (0.01-0.27)
33	21 (4.2)	1.15 (0.71-1.75)	10 (3.6)	0.47 (0.23-0.87)	4 (8.0)	0.15 (0.04-0.38)	3 (10.3)	0.11 (0.02-0.32)
35	9 (1.8)	0.49 (0.22-0.93)	3 (1.1)	0.14 (0.03-0.41)	1 (2.0)	0.04 (0.00-0.21)	0	0.00 (0.00-0.14)
39	34 (6.9)	1.86 (1.29-2.59)	20 (7.1)	0.95 (0.58-1.46)	3 (6.0)	0.11 (0.02-0.33)	2 (6.9)	0.07 (0.01-0.27)
45	22 (4.4)	1.20 (0.75-1.82)	6 (2.1)	0.28 (0.10-0.62)	1 (2.0)	0.04 (0.00-0.21)	0	0.00 (0.00-0.14)
51	109 (22.0)	5.95 (4.89-7.18)	57 (20.3)	2.70 (2.04-3.49)	7 (14.0)	0.26 (0.11-0.54)	4 (13.8)	0.15 (0.04-0.38)
52	61 (12.3)	3.33 (2.55-4.28)	35 (12.5)	1.66 (1.15-2.30)	4 (8.0)	0.15 (0.04-0.38)	2 (6.9)	0.07 (0.01-0.27)
56	44 (8.9)	2.40 (1.75-3.23)	19 (6.8)	0.90 (0.54-1.40)	5 (10.0)	0.19 (0.06-0.44)	3 (10.3)	0.11 (0.02-0.32)
58	22 (4.4)	1.20 (0.75-1.82)	14 (5.0)	0.66 (0.36-1.11)	7 (14.0)	0.26 (0.11-0.54)	2 (6.9)	0.07 (0.01-0.27)
59	26 (5.2)	1.42 (0.93-2.08)	1 (0.4)	0.05 (0.00-0.26)	0	0.00 (0.00-0.14)	0	0.00 (0.00-0.14)
66	46 (9.3)	2.51 (1.84-3.35)	24 (8.5)	1.14 (0.73-1.69)	4 (8.0)	0.15 (0.04-0.38)	1 (3.5)	0.04 (0.00-0.21)
68	37 (7.5)	2.02 (1.42-2.78)	17 (6.1)	0.80 (0.47-1.29)	4 (8.0)	0.15 (0.04-0.38)	3 (10.3)	0.11 (0.02-0.32)
At least 1 oncogenic type	399 (80.4)	21.79 (19.70-24.03)	232 (82.6)	10.97 (9.61-12.48)	50 (100.0)	1.87 (1.39-2.46)	29 (100)	1.07 (0.72-1.54)
**Non-oncogenic HPV types**								
6	74 (14.9)	4.04 (3.17-5.07)	21 (7.5)	0.99 (0.61-1.52)	3 (6.0)	0.11 (0.02-0.33)	1 (3.5)	0.04 (0.00-0.21)
11	9 (1.8)	0.49 (0.22-0.93)	1 (0.4)	0.05 (0.00-0.26)	2 (4.0)	0.07 (0.01-0.27)	0	0.00 (0.00-0.14)
34	7 (1.4)	0.38 (0.15-0.79)	1 (0.4)	0.05 (0.00-0.26)	0	0.00 (0.00-0.14)	0	0.00 (0.00-0.14)
40	16 (3.2)	0.87 (0.50-1.42)	1 (0.4)	0.05 (0.00-0.26)	2 (4.0)	0.07 (0.01-0.27)	1 (3.5)	0.04 (0.00-0.21)
42	7 (1.4)	0.38 (0.15-0.79)	0	0.00 (0.00-0.17)	0	0.00 (0.00-0.14)	0	0.00 (0.00-0.14)
43	27 (5.4)	1.47 (0.97-2.14)	3 (1.1)	0.14 (0.03-0.41)	0	0.00 (0.00-0.14)	0	0.00 (0.00-0.14)
44	4 (0.8)	0.22 (0.06-0.56)	1 (0.4)	0.05 (0.00-0.26)	0	0.00 (0.00-0.14)	0	0.00 (0.00-0.14)
53	56 (11.3)	3.06 (2.31-3.97)	28 (10.0)	1.32 (0.88-1.91)	3 (6.0)	0.11 (0.02-0.33)	2 (6.9)	0.07 (0.01-0.27)
54	16 (3.2)	0.87 (0.50-1.42)	8 (2.9)	0.38 (0.16-0.75)	3 (6.0)	0.11 (0.02-0.33)	2 (6.9)	0.07 (0.01-0.27)
70	6 (1.2)	0.33 (0.12-0.71)	6 (2.1)	0.28 (0.10-0.62)	1 (2.0)	0.04 (0.00-0.21)	0	0.00 (0.00-0.14)
74	13 (2.6)	0.71 (0.38-1.21)	6 (2.1)	0.28 (0.10-0.62)	0	0.00 (0.00-0.14)	0	0.00 (0.00-0.14)
Only non-oncogenic type	97 (19.6)	5.30 (4.29-6.46)	49 (17.4)	2.32 (1.71-3.06)	0	0.00 (0.00-0.14)	0	0.00 (0.00-0.14)

^1^Number of person-years: 1831.46 (HPVI); 2114.51 (6MPI); 2676.71 (CIN1+); 2703.47 (CIN2+).

^2^ N = 982.

Some women experienced concomitant HPV infections, concomitant CIN lesions and/or CIN lesions associated with more than 1 HPV type. Therefore the number of observations for each individual HPV type do not add up to the total number of women experiencing an infection or lesion (N).

6MPI: 6-month persistent HPV infection; CI: confidence interval; CIN: cervical intraepithelial neoplasia; HPV: human papillomavirus; HPVI: HPV infection of any duration; PY: person-years.

Among oncogenic HPV types, the most commonly detected HPVIs were HPV-51 (5.95 per 100 PY), HPV-16 (4.91 per 100 PY), HPV-52 (3.33 per 100 PY), HPV-18 (2.73 per 100 PY), and HPV-66 (2.51 per 100 PY) (Table 1). These types were also the most commonly detected 6MPIs, although HPV-18 was slightly less common than HPV-66 (Table 1). HPV-6 and HPV-53 were the most common non-oncogenic HPV types detected in HPVI (4.04 and 3.06 per 100 PY, respectively) and 6MPI (0.99 and 1.32 per 100 PY, respectively) (Table 1). The incidence of infection with at least one oncogenic HPV type was considerably higher than the incidence of infection with only non-oncogenic types: 21.79 versus 5.30 per 100 PY, respectively, for HPVI, and 10.97 versus 2.32 per 100 PY, respectively, for 6MPI (Table 1; Figure 2a and b).

In the multivariable analysis, the main covariate associated with increased risk of acquiring an HPVI was a higher number of lifetime sexual partners. The hazard ratio [HR] was 4.76 (95% CI: 3.82-5.95) and 19.22 (14.02-26.34) for 2-3 partners and ≥4 partners, respectively (Table 2). Other covariates significantly associated with increased risk were history of infection with *Chlamydia trachomatis* (HR: 3.00 [2.07-4.36]) and cumulative duration of hormone use (HR: 2.18 [1.68-2.83] for 1-12 months, 3.26 [2.45-4.34] for 13-48 months and 5.42 [2.84-10.36] for >48 months). Being a single woman was significantly associated with an increased risk of acquiring an HPVI (HR: 1.45 [1.04-2.04]) compared with living with a partner currently or in the past. Tobacco exposure did not increase the risk in the multivariable analysis, although it was significant in the univariate analysis (HR: 1.66 [1.33-2.08], p < 0.0001).

**Table 2:** **Factors associated with risk of the first cervical HPVI or 6MPI (multivariable Cox proportional-hazards analysis)**

Covariate	Category	HPVI				6MPI			
		No. of women (N = 982)	No. of women with HPVI (N = 496)	HR (95% CI)	p-value	No. of women (N = 982)	No. of women with 6MPI (N = 281)	HR (95% CI)	p-value
Region	Europe	644	345	1		644	201	1	
	Asia Pacific	168	58	1.11 (0.81-1.52)	0.5055	168	36	1.21 (0.78-1.86)	0.3995
	Latin America	76	46	1.16 (0.84-1.60)	0.3627	76	24	0.99 (0.63-1.57)	0.9773
	North America	94	47	0.88 (0.64-1.20)	0.4273	94	20	0.75 (0.47-1.20)	0.2283
				*0.5498*				*0.4934*	
Number of lifetime sexual partners^1^	1	800	234	1		800	127	1	
	2-3	430	190	4.76 (3.82-5.95)	<0.0001	470	111	4.19 (3.12-5.63)	<0.0001
	≥4	125	72	19.22 (14.02-26.34)	<0.0001	166	43	14.14 (9.35-21.37)	<0.0001
				*<0.0001*				*<0.0001*	
History of *Chlamydia trachomatis* during the past 12 months^1^	No	977	463	1		978	257	1	
	Yes	60	33	3.00 (2.07-4.36)	<0.0001	73	24	2.50 (1.57-4.00)	0.0001
				*<0.0001*				*0.0001*	
Cumulative duration of hormone use for contraception or other indication^1^	No use	804	105	1		804	59	1	
	1-12 months	727	173	2.18 (1.68-2.83)	<0.0001	746	111	2.10 (1.48-2.98)	<0.0001
	13-48 months	536	206	3.26 (2.45-4.34)	<0.0001	590	108	2.79 (1.91-4.09)	<0.0001
	>48 months	56	11	5.42 (2.84-10.36)	<0.0001	69	2	1.62 (0.39-6.74)	0.5107
				*<0.0001*				*<0.0001*	
Marital/partner status^1^	Living or lived with partner	238	42	1		271	19	1	
	Single	961	454	1.45 (1.04-2.04)	0.0291	963	262	1.75 (1.01-3.03)	0.0445
				*0.0291*				*0.0445*	
Tobacco exposure (number of pack-years)^1^	<0.5	862	402	1		862	223	1	
	≥0.5	153	94	1.17 (0.92-1.47)	0.1936	161	58	1.22 (0.90-1.66)	0.2085
				*0.1936*				*0.2085*	
Previous cervical HPV infection^1^	No	-	-	-	-	965	231	1	
	Yes	-	-	-	-	188	38	0.49 (0.33-0.72)	0.0003
				-				*0.0003*	

^1^Time-varying covariates. The number of women represents the number of observations that appeared at least once during the follow-up period. One woman can be counted in several categories. The number of events (HPVI or 6MPI) corresponds to the value recorded at the most recent visit before the endpoint.

Values in italics are the overall p-value.

Age at first sexual intercourse was not included in the multivariable analyses because p-values of >0.2 were obtained in the univariate analyses. The covariates of number of sexual partners during the past 12 months and hormone use or not for contraception or another indication were not included in the multivariable analyses because of overlap with the covariates of number of lifetime sexual partners and cumulative duration of hormone use or other indication, respectively.

6MPI: 6-month persistent HPV infection; CI: confidence interval; HPV: human papillomavirus; HPVI: HPV infection of any duration; HR: Hazard ratio.

The same covariates were significantly associated with higher risk of a 6MPI in the multivariable analysis, with the exception of hormone use for >48 months (Table 2). Being single was again significantly associated with higher risk of a 6MPI (HR: 1.75 [1.01-3.03]); previous cervical HPV infection was associated with lower risk (HR: 0.49 [0.33-0.72]) (Table 2).

### First cervical intraepithelial neoplasia grade 1 or greater and cervical intraepithelial neoplasia grade 2 or greater: incidence and association of covariates

Few cases of CIN1+ or CIN2+ were observed. A CIN1+ was detected in 50 women (5.1%; 1.87 per 100 person-years) (Table 1). No CIN1+ had been detected by the end of the follow-up period in 866 women (88.2%) who completed the study, and no CIN1+ had been detected by the time of their last visit in 66 women (6.7%) who discontinued before the end of the study. Corresponding values for CIN2+ were 29 (3.0%; 1.07 per 100 person-years), 886 (90.2%) and 67 (6.8%). The first cases of CIN1+ lesions were detected approximately 6 months following sexual initiation. The cumulative proportion of women developing a CIN1+ lesion was 3% at 24 months and 10% at 48 months (Figure 2c). The first cases of CIN2+ lesions were detected within approximately 12-18 months after sexual initiation; the cumulative proportion of women developing a CIN2+ lesion was 1% at 24 months and 6% at 48 months (Figure 2d). CIN lesions were most commonly associated with HPV-16 (Table 1; Figure 2c and d). Other oncogenic HPV types most often detected in a CIN lesion were HPV-51, HPV-58, and HPV-31. No lesions associated only with non-oncogenic HPV types were found (Table 1).

The covariate most strongly associated with increased risk of CIN1+ in the multivariable analysis was previous cervical HPV infection (HR: 35.70 [95% CI: 8.47-150.50]) (Table 3). Having ≥4 lifetime sexual partners was also significantly associated with increased risk (HR: 5.60 [2.28-13.77]). Cumulative duration of hormone use was associated with higher risk, but just failed to achieve statistical significance (HR: 3.61 [0.94-13.86], 3.84 [1.00-14.77] and 6.09 [0.89-41.63] for 1-12, 13-48 and >48 months, respectively). Being from the Asia-Pacific region was significantly associated with increased risk (HR: 2.99 [1.03-8.70]), although it should be noted that only six women from this region experienced CIN1 +.

**Table 3:** **Factors associated with risk of the first CIN1+ or CIN2+ (multivariable Cox proportional-hazards analysis)**

Covariate	Category	CIN1+				CIN2+			
		No. of women (N = 982)	No. of women with CIN1+ (N = 50)	HR (95% CI)	p-value	No. of women (N = 982)	No. of women with CIN2+ (N = 29)	HR (95% CI)	p-value
Region	Europe	644	35	1		644	21	1	
	Asia Pacific	168	6	2.99 (1.03-8.70)	0.0441	168	3	0.76 (0.20-2.93)	0.6942
	Latin America	76	3	1.18 (0.34-4.05)	0.7932	76	1	0.57 (0.07-4.37)	0.5869
	North America	94	6	1.93 (0.80-4.68)	0.1439	94	4	1.73 (0.58-5.18)	0.3238
				*0.1480*				*0.6452*	
Age at first sexual intercourse	≥18 years	801	41	1		801	26	1	
	15-17 years	181	9	0.46 (0.21-1.02)	0.0545	181	3	0.18 (0.05-0.64)	0.0086
				*0.0545*				*0.0086*	
Number of lifetime sexual partners^1^	1	800	9	1		800	5	1	
	2-3	537	15	1.73 (0.72-4.15)	0.2213	537	10	1.93 (0.63-5.92)	0.2529
	≥4	245	26	5.60 (2.28-13.77)	0.0002	248	14	4.02 (1.24-13.05)	0.0203
				*0.0001*				*0.0504*	
Cumulative duration of hormone use for contraception or other indication^1^	No use	804	3	1		Not included			
	1-12 months	786	13	3.61 (0.94-13.86)	0.0614				
	13-48 months	707	32	3.84 (1.00-14.77)	0.0502				
	>48 months	102	2	6.09 (0.89-41.63)	0.0653				
				*0.2157*					
Tobacco exposure (number of pack-years)^1^	<0.5	862	36	1		Not included			
	≥0.5	177	14	1.44 (0.76-2.74)	0.2622				
				*0.2622*					
Previous cervical HPV infection^1^	No	974	2	1		975	1	1	
	Yes	431	48	35.70 (8.47-150.50)	<0.0001	432	28	42.30 (5.61-318.63)	0.0003
				*<0.0001*				*0.0003*	

^1^Time-varying covariates. The number of women represents the number of observations that appeared at least once during the follow-up period. One woman can be counted in several categories. The number of events (CIN1+ or CIN2+) corresponds to the value recorded at the most recent visit before the endpoint.

Values in italics are the global p-value.

History of *Chlamydia trachomatis* (CIN1+ and CIN2+), cumulative duration of hormone use (CIN2+), marital/partner status (CIN1+ and CIN2+) and tobacco exposure (CIN2+) were not included in the multivariable analyses because p-values of >0.2 were obtained in the univariate analyses. The covariates of number of sexual partners during the past 12 months and hormone use or not for contraception or another indication were not included in the multivariable analyses because of overlap with the covariates of number of lifetime sexual partners and cumulative duration of hormone use or other indication, respectively.

CI: confidence interval; CIN: cervical intraepithelial neoplasia; HPV: human papillomavirus; HR: hazard ratio.

Previous cervical HPV infection also had the greatest influence on risk of developing CIN2+, with a HR of 42.30 (5.61-318.63), and having ≥4 lifetime sexual partners was also associated with higher risk (HR: 4.02 [1.24-13.05]) (Table 3). Age 15-17 years at first sexual intercourse was significantly associated with lower risk of developing CIN2+ compared with age ≥18 years; however, only 3 women with first experience of sexual intercourse at age 15-17 years experienced a CIN2 +.

A sensitivity analysis not including the factor of previous infection in the model did not change the HR for the other covariates for CIN1+ and CIN2+ (data not shown). Likewise, a sensitivity analysis of all lesions (including both HPV-positive and HPV-negative cases) revealed similar results except for CIN2+ where the cumulative duration of hormone use had a univariate p-value <0.2 and was thus included in the multivariable analysis (whereas it was not included in the multivariable analysis of HPV-positive CIN2+). However, it was not significant in the multivariable analysis.

## Discussion

The analysis confirmed that HPV infection may be acquired relatively quickly following first sexual intercourse. The first cases of HPV infections were detected within 2-3 months following sexual initiation, and two thirds of women had acquired an infection within the 48 month follow-up period. Persistent infections were less common, detected in approximately one-quarter of women. The first cases of CIN1+ lesions were detected approximately 6 months following sexual initiation, and the first CIN2+ lesions approximately 12-18 months, but both were experienced by very few women.

Other studies of virginal women experiencing sexual intercourse for the first time have shown broadly similar rates of oncogenic HPV acquisition, although it is difficult to compare estimates directly because of differences across studies in outcomes and follow-up times. At 2 years after sexual initiation, we found an incidence of HPV infection of 44%. Two previous studies have found slightly lower incidences at this time point. A population-based cohort study conducted in Denmark found an incidence of 35% among 70 women recruited as virgins and experiencing first sexual intercourse within the follow-up period [15], whilst a study of 444 HPV DNA-negative female students from the US (94 recruited as virgins and experiencing first sexual intercourse within the follow-up period) found an incidence of 39% for both women who were virgin and those who were sexually active at enrolment [13]. In the latter study, the minimum time between first sexual intercourse and detection of HPV was less than 1 month [13]. Likewise, the incidence of HPV infection at 3 years was 53% in our analysis, slightly higher than the 46% incidence at the same time point found among 242 women from the UK recruited within 6 months of first sexual intercourse and with only one partner [14]. In a study of 206 women from the Guanacaste cohort recruited as virgins and experiencing first sexual intercourse within the follow-up, the incidence of cervical HPV infection was 53% after a median follow-up of 3.6 years [16], compared with an incidence of 62% after 4 years in our analysis.

In our analysis, the most common oncogenic HPV types in HPVI and 6MPI were HPV-16 and HPV-51, followed by HPV-52, and then by HPV-18 and HPV-66. HPV-6 was the most common non-oncogenic type; however, its incidence may be underestimated because the study did not systematically evaluate the external genital tract. In the overall PATRICIA cohort, the most common prevalent HPV types at study entry were HPV-16, followed by HPV-51, HPV-18, and HPV-31 [22]. This is in line with previous studies of the occurrence of different HPV types, although there is considerable variation between studies and regions in the exact ranking and incidence of the different types, and 95% confidence intervals of incidence are broad. In the Guanacaste subcohort of women recruited as virgins, the most common oncogenic HPV types detected were HPV-16, HPV-66, and HPV-52; HPV-53 was the most common non-oncogenic type detected [16]. Amongst HPV DNA-negative students from the US, the most common HPV types detected at first infection were HPV-16, HPV-56, and HPV-6 [13]. A meta-analysis of more than 1 million women worldwide found HPV-16 to be by far the most common type in women with normal cytology, followed by HPV-18, HPV-52, HPV-31, and HPV-58 [23].

Few women experienced CIN1+ and even fewer experienced CIN2+, so estimates of time to lesion development and the most common HPV type associated with CIN lesions have somewhat low precision. However, it was clear that HPV-16 was by far the most frequent type found in CIN1+ and CIN2+, as seen in other studies [24],[25].

In our analysis, higher number of lifetime sexual partners was most strongly associated with a higher risk of an HPVI or 6MPI. History of *Chlamydia trachomatis* infection and longer cumulative duration of hormone use also significantly increased the risk of acquisition. Although the univariate analysis indicated an increased risk for HPVI with tobacco exposure, the multivariable analysis showed no significant association. For the overall PATRICIA cohort, infection with HPV at entry was significantly associated with not being married or living with a partner, tobacco exposure, age <15 years at first sexual intercourse, higher number of sexual partners during the past 12 months, longer duration of hormone use, and history of a sexually transmitted infection [22]. The PATRICIA findings are in line with previous studies of risk factors influencing acquisition of HPV infection. In the Danish study of women experiencing first sexual intercourse mentioned previously, having ≥3 sexual partners increased the risk of infection 9-fold; age, smoking habits, oral contraceptive use, and age at first sexual intercourse were not significantly associated with infection [15]. In the US study of students, current smoking, current oral contraceptive use, higher number of lifetime sexual partners, male partners’ number of prior sexual partners, and knowing a partner for less than 8 months before sexual intercourse were predictors of HPV infection [13].

Previous cervical HPV infection was associated with lower risk of a 6MPI with any HPV type, perhaps indicating a protective effect of naturally acquired antibodies to the prior infection. In contrast, previous cervical HPV infection was strongly associated with increased risk of developing CIN1+ and CIN2+. In another analysis of the whole PATRICIA control group, previous infection with an oncogenic HPV type was also significantly associated with increased risk of developing CIN1+ and CIN2+ [17]. Accumulation of HPV infections may indicate that an individual's immune system may have limited ability to clear infections, thus increasing their risk of developing CIN. In contrast to our findings, some studies have shown that there is a higher chance of acquiring a new HPV type if already infected [26]-[28]. Unfortunately, in the PATRICIA trial, information on HPV serology over time was only available for a sub-cohort of women, and the number of women in the control group of the immunogenicity cohort who had not initiated sexual intercourse at enrolment was too small for meaningful analysis to explore the role of serology in the risk of HPV infections and CIN.

Four or more lifetime sexual partners was also associated with increased risk of CIN1+ and CIN2+. An association between cervical cancer and higher number of sexual partners has been seen in previous studies [8]. Younger age at first sexual intercourse was associated with lower risk of CIN2+; however, as there were only three cases of CIN2+ in the 15-17 year age group, this finding should be interpreted cautiously. Younger age at first sexual intercourse has been previously shown to be associated with increased risk of cervical cancer [8].

A strength of our analysis is that participants of the PATRICIA study were well-characterized, with high follow-up rates over 4 years and standardized virologic and histologic sampling methods. Although the population of the present analysis was a relatively small subset of the entire PATRICIA population, more women were included in this analysis than in any other study of virginal women. Study limitations included the fact that women from Finland made up approximately two thirds of the population who had not had experience of sexual intercourse before enrolment, a consequence of the school-based trial recruitment strategy in Finland. In addition, PATRICIA included only women aged 15-25 years with no history of immunosuppressive disease, limiting its generalizability. The smaller population in the present analysis meant that the 4-year follow-up period was long enough to detect only a small number of CIN cases, limiting the conclusions that can be drawn about the time between first sexual intercourse and development of pre-cancerous lesions.

## Conclusion

Infection with HPV can occur within 2-3 months following first sexual intercourse, and the first CIN1+ lesions within approximately 6 months. The role of previous cervical HPV infection in increasing the risk of developing a pre-cancerous lesion is confirmed, as is the role of the number of lifetime sexual partners in acquisition of an HPV infection. Our estimates of time to infection and time to CIN after sexual initiation are important as they will inform modeling exercises on the natural history of cervical carcinogenesis and the impact of HPV vaccination programs. Furthermore, without underestimating the value of vaccination at older ages, our findings emphasize the importance of vaccinating at a young age, well before girls begin sexual activity, most likely achieved via school-based vaccination programs.

## Additional file

## Electronic supplementary material

Additional file 1: List of Independent Ethics Committees/ Institutional Review Boards. (DOC 572 KB)

Below are the links to the authors’ original submitted files for images.

Authors’ original file for figure 1

Authors’ original file for figure 2

Authors’ original file for figure 3

Authors’ original file for figure 4

Authors’ original file for figure 5
