# Supplementary material for: Risk of first cervical HPV infection and pre-cancerous lesions after onset of sexual activity: analysis of women in the control arm of the randomized, controlled PATRICIA trial
Source: BMC Infect Dis. 2014 Oct 30;14:551. doi: 10.1186/s12879-014-0551-y (PMC4251672; doi:10.1186/s12879-014-0551-y)
Supplement: Supplementary file 1 — Additional file 1: List of Independent Ethics Committees/ Institutional Review Boards. (DOC 572 KB) [file 12879_2014_551_MOESM1_ESM.doc]

# Appendix 1

**List of Independent Ethics Committees/ Institutional Review Boards**

| **Centre Number(s) *** | **Ethics Review Body** | **Location** |
| --- | --- | --- |
| 004538 | Royal Women’s Hospital  132 Grattan Street-Carlton 3053 | AUSTRALIA |
| 004539 | Alfred Human Research Ethics Committee  Alfred Hospital-Commercial Road  Melbourne 3004 |
| 004980 | Southern Tasmania Health and Medical Research Ethics Committee  University of Tasmania  Research and Development Office  Private Bag 01-Hobart Tasmania 7001 |
| 004981 | King Edward Memorial Hospital for Women  374 Bagot Rd Subiaco WA 6008  St John of God Healthcare  175 Cambridge St Subiaco WA 6008 |
| 004993 | Royal Adelaide Hospital Research Ethics Committee  Level 3 Hansen Centre  Rpoyal Adelaide Hospital North terrace  Adelaide SA 5000 |
| 005152 | Sydney West Area Health Service Human Research Ethics Committee  Westmead Hospital  Westmead NSW2145 |
| 004552 | Commissie voor Medische Ethiek  Universitair Ziekenhuis Antwerpen (UZA)  Wilrijkstraat 10-2650 Edegem | BELGIUM |
| 004553 | Commissie Medische Ethiek  Akademisch Ziekenhuis-VUB  Laarbeeklaan 101-1090 Brussels |
| 004556 | Commissie Medische Ethiek  Klinisch Onderzoek-Faculteit Geneeskunde  Herestraat 49-30Leuven |
| 005013 | Comitê de Ética em Pesquisa  Faculdade de Ciências Médicas - UNICAMP  Cidade Universitária Zeferino vaz  Rua Tessalia Vieira de Camargo, 126 - caixa postal: 6111  13083-970 - Campinas / SP - Brasil | BRAZIL |
| 005014 | Comitê de Ética em Pesquisa em Seres Humanos do Hospital de  Clínicas da Universidade Federal do Paraná  Rua General Carneiro, 181  80060-160 - Curitiba / PR |
| 005017 | Comitê de Ética do Hospital de Clínicas de Porto Alegre  Rua Ramiro Barcelos, 2350 - 2o andar - Bonfim  90035-003 - Porto Alegre / RS |
| 005548-005553-  005554-005555-  005556-14594 | Research Review Board Inc.  119 University Avenue East  Waterloo ON, N2J 2W1 | CANADA |
| 005549 | Biomedical Ethics research Board  University of Manitoba  P126-770 Bannatyne Avenue  Winnipeg MB, R3E0W3 |
| 005550 | Research Ethics Committee  Sir Mortimer B.Davis Jewish General Hospital  3755 Cote Ste Catherine Road a-925  MONTREAL QC, H3T 1E2 |
| 005551 | Comité d.Ethique de la Recherche Clinique CHUL  Centre Hospitalier Universitaire de Quebec . CHUL  Laboratoire et Service d.Infectiologie  2705 Boul Laurier local S-745E  Sainte Foy QC, G1V 4G2 |
| 005552 | Health research Ethics Board  University of Alberta  213 Heritage Medical Research Building  Edmonton AB T6G 2S2 |
| 004880-04881-  04882-04885-  04886-04887-  04888-04889-  04890-04891-  04892-04893-  09461-12292-  12293-12294-  12296-12297-15982 | ETENE-Lääketieteellinen tutkimuseettinen jaosto  Sosiaali- ja terveysministeriö  Kirkkokatu 4  00170 Helsinki | FINLAND |
| 004322-04551-  10833-11270-  11271-11272-  11273-11274-  11276-11277-  11278-11279-  11280-11281-  11282-11284-  12115 | Ethikkommission der Bayerischen Landesärztekammer  Mühlbaurstr 16-81677 Munchen | GERMANY |
| 004515 | Comitato Etico Provinciale di Modena  Policlinico di Modena Azienda Ospedaliera  Via del Pozzo 71-41100 Modena | ITALY |
| 004516 | Comitato etico della Fondazione IRCCS  Ospedale Maggiore Policlinico Mangiagalli e Regina Elena  Via F Sforza 35 -20112, Milan |
| 5033 | Comisión Nacional de Investigación Cientifica y Subcomision de Etica  del Instituto Mexicano del Seguro Social  4°piso Edificio B Unidad de Congresos  Avenida Cuahtemoc 330 Colonia Doctores Mexico DF 06725 | MEXICO |
| 004425 | The Ethics Committee  San Pablo Colleges Medical centre  Barangay San Rafael Maharlika Highway  4000 San Pablo City Laguna | PHILIPPINES |
| 005114 | Institutional Review Board  Makati Medical Center  2 Amorsolo street Makati City |
| 005116 | De La Salle health Sciences Campus Ethics Rreview Board  (DLS-HSC-ERB)  De La Salle Angelo King Medical Research Center  Congressional Avenue, Dasmarinas Cavite 4114 |
| 005692 | Institutional Review Board  Calamba Medical Center . Crossing Calamba Laguna |
| 009976 | Ethics Review Board-Medical Arts Building  University of Perpetual Help Medical Center  Alabang-Zapote Road- Las Pinas City |
| 009977 | Committee on research Implementation and development and review  Board  Healthserv-Los Banos Inc,8817 National Highway  Los Banos Laguna |
| 009978 | Research Implementation and Development Office  College of Medicine  University of the Philippines taft Avenue Manila 1000 |
| 004569 | Comite Etico de Investigacion Clinica Del Hopsital Clinic I Provincial de  Barcelona  C/Villarroel 170 . 08036 Barcelona | SPAIN |
| 004570 | Comité Etico de Investigacion Clinica Hospital San Carlos  Martin Lagos s/n-28040 Madrid |
| 004572 | Comité Etico de Investigacion Clinica Hospital Vall D.Hebron  Passeig de la Vall d.Hebron 119-129 08035 Barcelona |
| 005279 | Comite Etico De Investigacon Clinica Del Hospital De Mostoles  C/Rio Jucar s/n 28935 Mostoles madrid |
| 005280 | Comite Etico De Investigacion Clinica Ciudad Sanitaria de Bellvitge  Feixa Llarga s/n  08907 L.Hospitalet de Llobregat |
| 005281 | Comite Etico de Investigacion Clinica  Hospital Universitario de La Princesa  C/Diego d Leon 62 Planta 9a Sector 2-28006 Madrid |
| 4927, 014244, 4955,  4951, 4952, 013831,  4923, 14689, 14690,  4925, 014245, 4928,  8233, 4931, 9039,  5475, 4934, 013833,  4940, 4945, 8231,  13835, 4947,  014243, 013834,  4944, 4943, 4937,  9066, 9068, 4939 | Quorum Review IRB 1601 Fifth Ave., Suite 1000  Seattle, WA 98101 | US |
| 4930 | Local/University of Minnestota  IRB  Mayo Mauil Code 820  D-528 Mayo Memorial Bldg  420 Delaware Stret S. E.  Minneapolis, MN 55455 |
| 4932 | Local/Medical College of Georgia 1120 15th Street CJ-2103  Augusta, GA 30912 |
| 4933 | Local/Univ. of Louisville  MedCenter One, Suite 200  501 E. Broadway  Louisville, KY 40202-1798 |
| 4920 | Western Institutional Review Board  3535 Seventh Ave, SW  Olympia, WA 98502 |
| 4946 | Hiawaii Pacific Health Res. Institute  1100 Ward Ave.  Suite 1045  Honolulu, Hawaii 96814 |
| 14460 | University of Oklahoma  Office of Research Administration Stanton Young  Blvd., LIB 121  Oklahoma City, OK 73117 |
| 4938 | Local/IRB for Health Scoences Research  PO Box 800483  University of Virginia  Charlottesville, VA 22908 |
| 4971 | Local/Morristown Atlantic Health Sys.  IRB  95 Madison Ave.  Morristown, NJ 07960 |
| 4958 | Biomedical Research Alliance of New York, LLC  Institituional Review Board  225 Community Drive, Suite 100 Great Neck, NY 11021 |
| 4949 | Local/Human Subjects Office  340 Medicine Administration Bldg  Iowa City, Iowa 52242-1101 |
| 4918 | Local/The MetroHealth System IRB  MetroHealth Medical Center  2500 MetroHealth Dr.  Cleveland, OH 44109-1998 |
| 4941 | Local/University of Miami  1500 NW 12 Ave Suite 1000  Miami, FL 33136 |
| 4929 | Local/  Human Research Review Committee  MSC 08 4560 BMSB Room B71  1 University Of New Mexico  Albuquerque, NM 87131-0001 |
| 004543 | Ethics Committee  National Taiwan University Hospital  7 Chung Shan South Road Taipei 100 | TAIWAN |
| 004544 | Tri-Service General Hospital Institutional Review Board  N°325,Sec 2, Cheng-gong Road, Neihu District 114 Taipei |
| 011406 | Taipei Veterans General Hospital Institutional review Board  N° 201, Sec 2, Shih-Pai Road Taipei 112 |
| 003891 | Ethical Review Committee  Royal Thai Army Medical Department  315 Rajavithi Road, Rajathevee Bangkok 10400 | THAILAND |
| 003892 | Research Affairs  Faculty of Medicine Chulalongkorn University  1873 Rama Itumwan Bangkok 10330 |
| 003893 | Ethics Coimmittee Faculty of Medicine Siriraj Hospital Mahidol  University  2 Pranok Road Bangkoknoi Bangkok 10700 |
| 04859-04861-  04894-14372-14373 | NorthWest Multi Centre Research Ethics Committee  Gateway House  Picadilly South, Manchester M60 7LP | UK |
| 5051 | Grampian LREC  Grampian NHS Board  Summerfield House, 2 Eday Road, Aberdeen, AB15 6RE |
| 4859 & 14373 | Central Manchester LREC  Room 181, 1st Floor, Gateway House  Piccadilly South, Manchester, M60 7LP |
| 4894 | Camden & Islington Community LREC  Room 3/14, 3rd Floor, West Wing, St Pancras Hospital  4 St Pancras Way, London, NW1 0PE |

* GSK Biologicals assigned centre number
