# Supplementary figures and images for: Risk of first cervical HPV infection and pre-cancerous lesions after onset of sexual activity: analysis of women in the control arm of the randomized, controlled PATRICIA trial
Source: BMC Infect Dis. 2014 Oct 30;14:551. doi: 10.1186/s12879-014-0551-y (PMC4251672; doi:10.1186/s12879-014-0551-y)

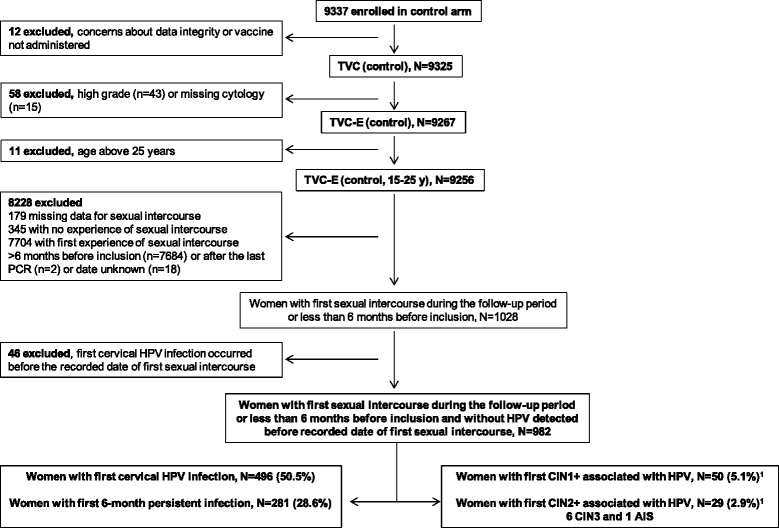

Supplement: Supplementary file 2 — Authors’ original file for figure 1 [file 12879_2014_551_MOESM2_ESM.gif]

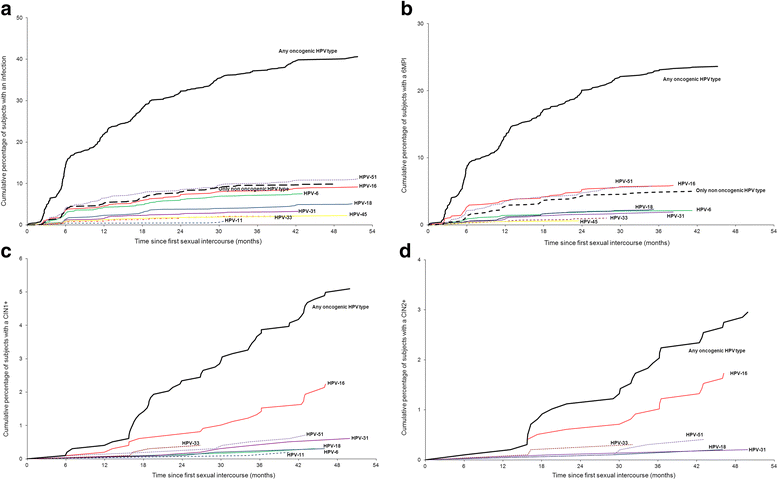

Supplement: Supplementary file 3 — Authors’ original file for figure 2 [file 12879_2014_551_MOESM3_ESM.gif]

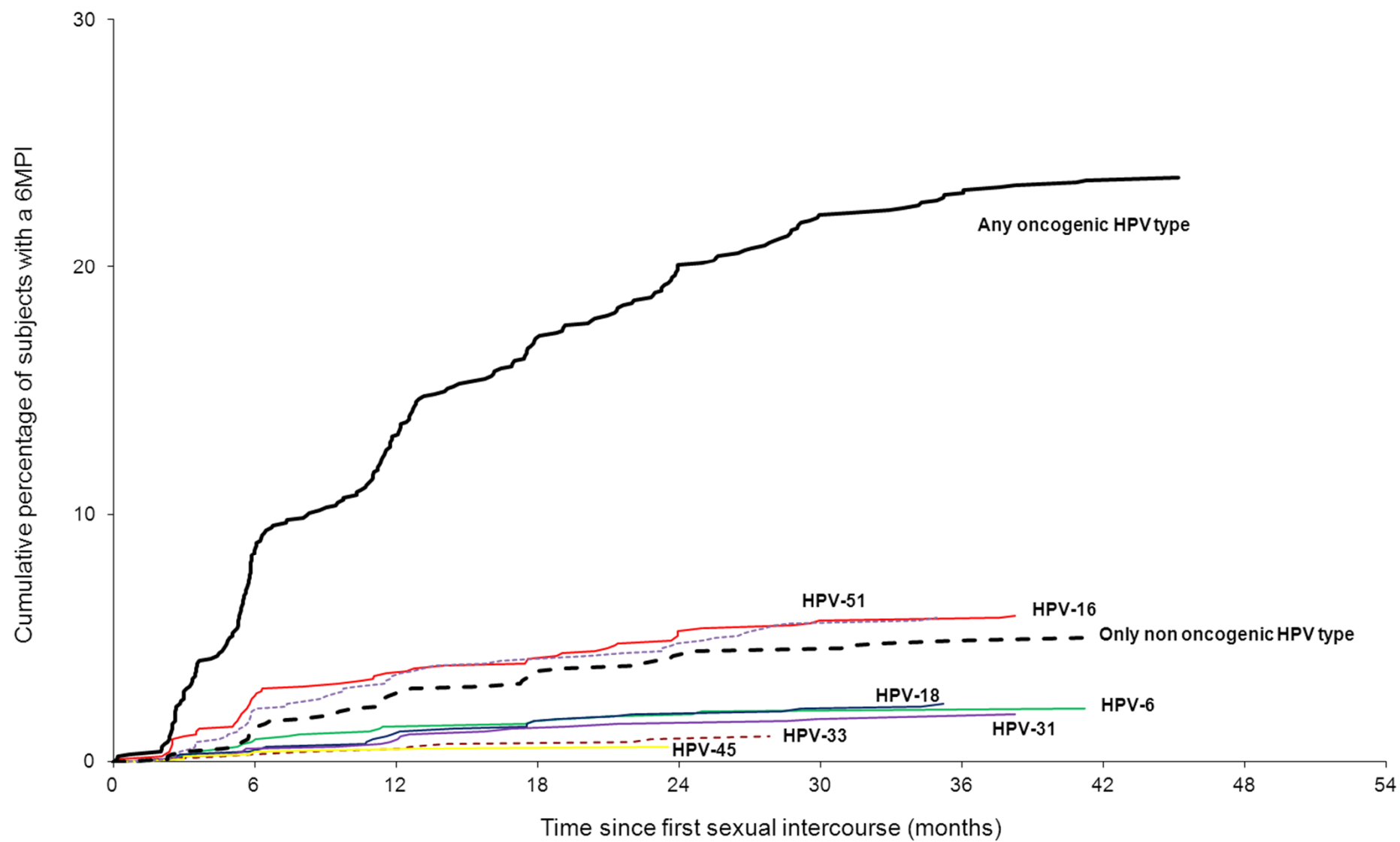

Supplement: Supplementary file 4 — Authors’ original file for figure 3 [file 12879_2014_551_MOESM4_ESM.pdf]

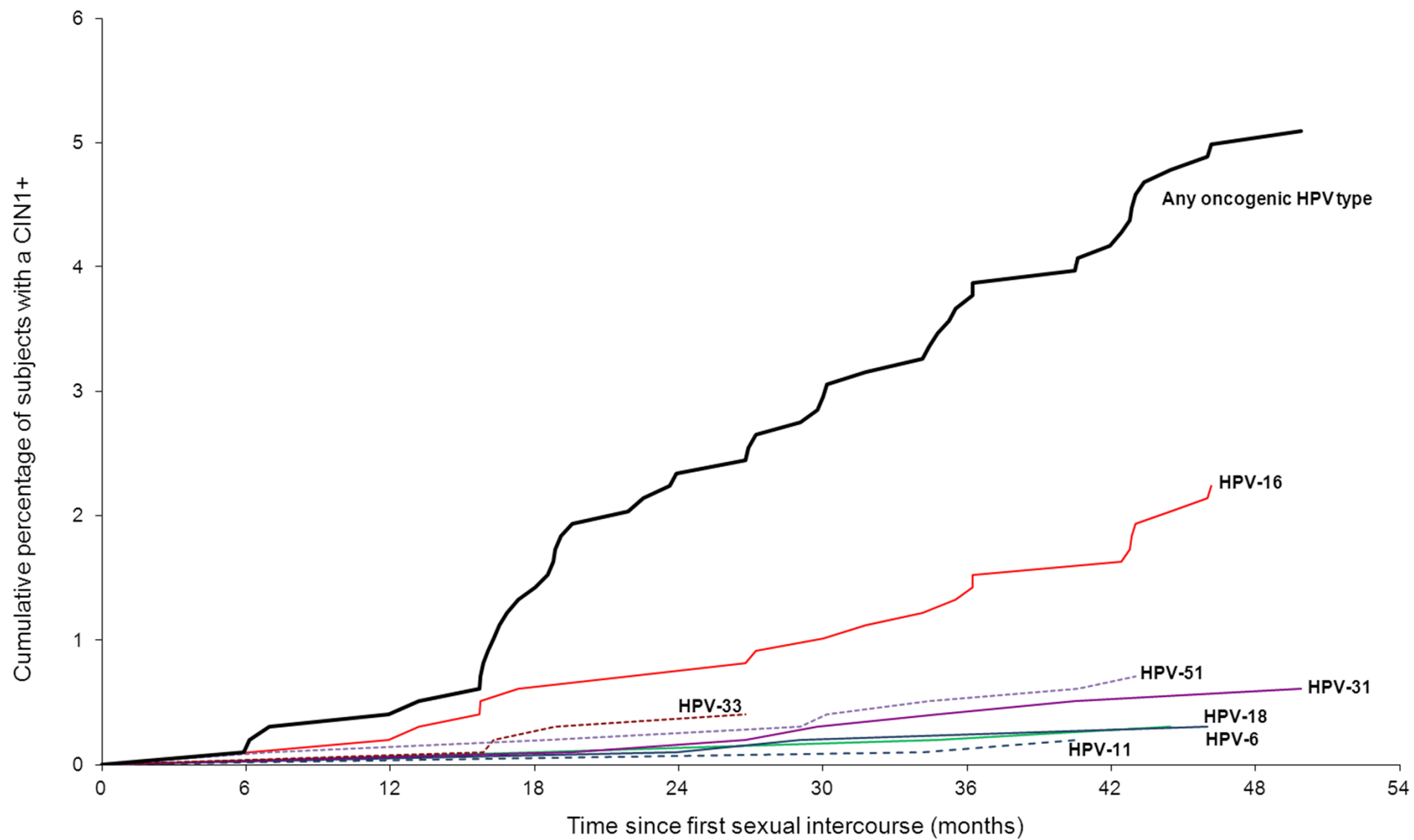

Supplement: Supplementary file 5 — Authors’ original file for figure 4 [file 12879_2014_551_MOESM5_ESM.pdf]

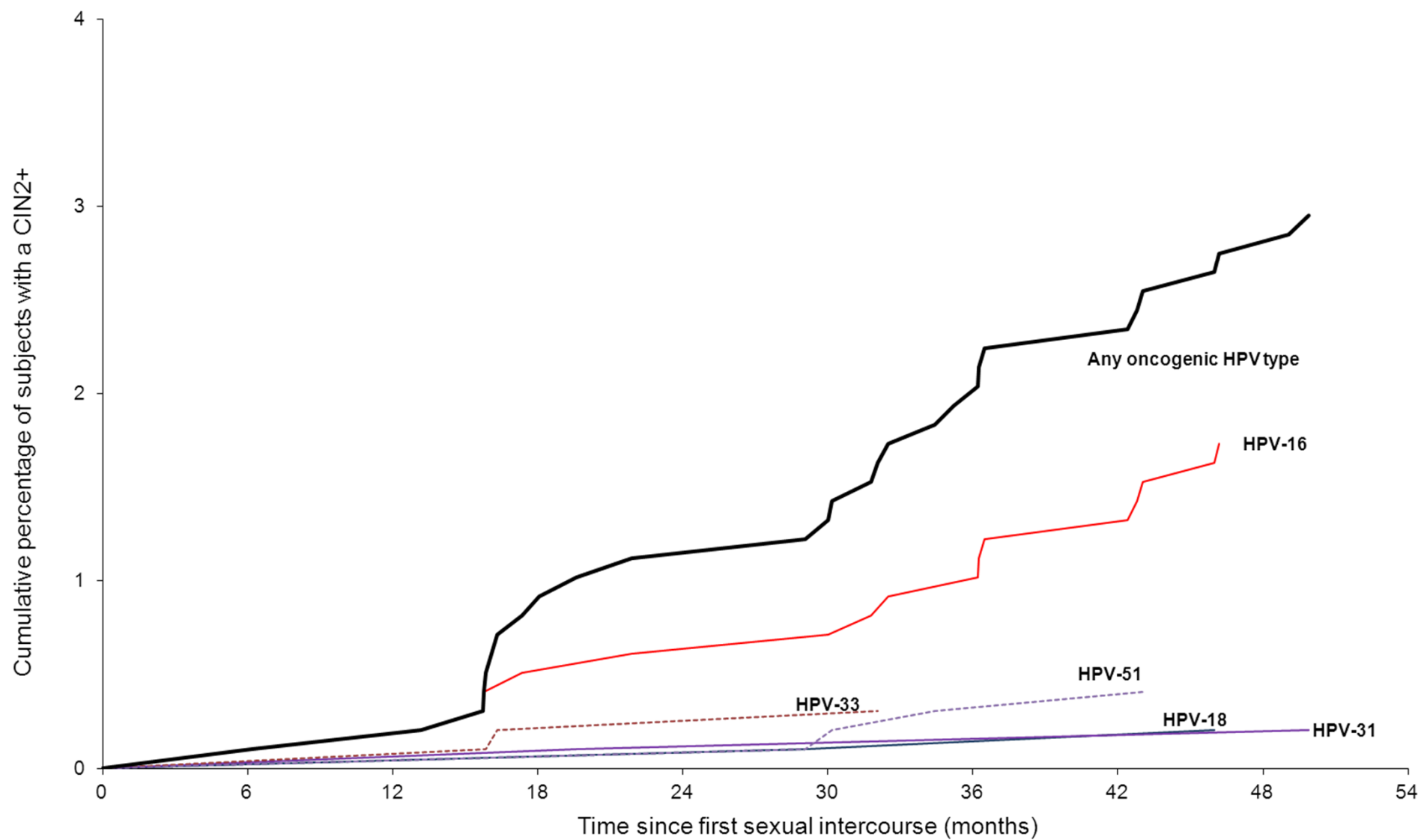

Supplement: Supplementary file 6 — Authors’ original file for figure 5 [file 12879_2014_551_MOESM6_ESM.pdf]
